# Initial steps towards a production platform for DNA sequence analysis on the grid

**DOI:** 10.1186/1471-2105-11-598

**Published:** 2010-12-14

**Authors:** Angela CM Luyf, Barbera DC van Schaik, Michel de Vries, Frank Baas, Antoine HC van Kampen, Silvia D Olabarriaga

**Affiliations:** 1 Bioinformatics Laboratory, Department of Clinical Epidemiology, Biostatistics and Bioinformatics, Academic Medical Center, PO Box 22700, 1100 DE Amsterdam, the Netherlands; 2 Virus Discovery Unit, Department of Medical Microbiology, Academic Medical Center, PO Box 22700, 1100 DE Amsterdam, the Netherlands; 3 Department of Neurogenetics, Academic Medical Center, PO Box 22700, 1100 DE Amsterdam, the Netherlands; 4 Biosystems Data Analysis, Swammerdam Institute for Life Science, University of Amsterdam, Nieuwe Achtergracht 166, 1018 WV Amsterdam, The Netherlands; 5 Netherlands Bioinformatics Centre, Geert Grooteplein 28, 6525 GA Nijmegen, the Netherlands

## Abstract

**Background:**

Bioinformatics is confronted with a new data explosion due to the availability of high throughput DNA sequencers. Data storage and analysis becomes a problem on local servers, and therefore it is needed to switch to other IT infrastructures. Grid and workflow technology can help to handle the data more efficiently, as well as facilitate collaborations. However, interfaces to grids are often unfriendly to novice users.

**Results:**

In this study we reused a platform that was developed in the VL-e project for the analysis of medical images. Data transfer, workflow execution and job monitoring are operated from one graphical interface. We developed workflows for two sequence alignment tools (BLAST and BLAT) as a proof of concept. The analysis time was significantly reduced. All workflows and executables are available for the members of the Dutch Life Science Grid and the VL-e Medical virtual organizations All components are open source and can be transported to other grid infrastructures.

**Conclusions:**

The availability of in-house expertise and tools facilitates the usage of grid resources by new users. Our first results indicate that this is a practical, powerful and scalable solution to address the capacity and collaboration issues raised by the deployment of next generation sequencers. We currently adopt this methodology on a daily basis for DNA sequencing and other applications. More information and source code is available via http://www.bioinformaticslaboratory.nl/

## Background

The next generation of DNA sequencers display a high increase in throughput. At the time of writing the Roche Titanium sequencer [1] produces 1,000,000 sequences that are about 420 nucleotides in length (3 GB of data) per sequence run, the Applied Biosystems Solid [2] generates 500,000,000 sequences of about 50-100 nucleotides in length (2-4 TB of data). Storing all the data is becoming a serious problem and the analysis becomes a time-consuming task. For some types of analysis (e.g., pair-wise comparison between all sequences of one sequence run) the computing time increases exponentially with the number of sequences. Also, researchers need to perform additional analyses that are not fully covered by standard packages (e.g., distributed by the sequencer vendors), requiring the development of new bioinformatics algorithms. At our hospital, we expect a wide variety and large number of DNA sequencing experiments due to the availability of two in-house high throughput sequencers. With the increase of the data size, number of experiments, and complexity of analyses, bioinformaticians are challenged with new problems in data management and data analysis. This is a problem that many other institutions are facing. We are therefore currently evaluating the use of grid and workflow technology to cope with challenges posed by bioinformatics analysis. This is motivated not only by the obvious needs of additional computing and storage capacity, but also by the wish to adopt more adequate technology to reuse and share analysis software and to more efficiently develop new analysis pipelines. Additionally, the collaborative nature of this domain, where researchers from several areas participate, also motivates the adoption of a distributed, open and federated framework enabled by grids. And finally, the BioAssist program [3] of the Netherlands Bioinformatics Centre (NBIC) [4] is set-ting up a bioinformatics platform for next generation DNA sequencing based on the Dutch Life Science Grid (LSGRID) [5], which is part of the Dutch Grid [6]. This infrastructure consists of computing and storage resources distributed among high performance computing organizations and research institutes, some of which take part in the European Grid Initiative (EGI) [7]. For all these reasons we were stimulated to explore the possibilities of grids to support DNA sequencing.

There are increasing efforts to apply grid infrastructures in bioinformatics. An early example from the DataGrid project is described in Jacq *et al *[8], more recently Craddock *et al *[9] described the annotation and classification of secreted proteins in bacterial genomes by combining web and grid services. Nevertheless, as described in Jacq *et al *[8], the implementation and use of such infrastructure is far from trivial. Adopting grid technology is complex for researchers that have limited experience with more complex infrastructures. It requires much awareness of (technical) grid details and exposure to (unfriendly) new user interfaces, e.g., command line utilities. Workflow and grid technology has already been deployed in the Medical Diagnosis and Imaging subprogram of the Virtual Laboratory for e-Science Project (VL-e) [10], where a user-friendly platform was developed to analyze functional Magnetic Resonance Imaging (fMRI) data on the Dutch Grid [11]. This platform is generic and can be adopted for other biomedical applications, being currently coined e-BioScience Infrastructure (e-BioInfra) [12].

In this paper we describe our initial steps to adopt grid and workflow technology for the analysis of data produced by high throughput DNA sequencers, evaluating if and how these technologies can be applied on a routine basis to improve our analysis capacity and facilitate collaboration with other users of the Dutch LSGrid.

## Methods

### Local infrastructure for sequence analysis

Next generation DNA sequencing gives a new impulse to applications such as whole genome (re-) sequencing, high throughput SNP analysis, splice variant detection and virus discovery. During and after the sequencing process, one has to rely on a good informatics infrastructure to store and analyze the data.

The images generated during the sequencing process are analyzed on the server/cluster attached to the DNA sequencer. The first step converts the information from images into sequences. In the rest of the document we will refer to this sequence data simply as 'data' or 'sequences'. Further analysis is performed with the software of the manufacturer of the sequencer, or alternatively (e.g. for non-standard studies) new algorithms are designed.

**Figure 1:**
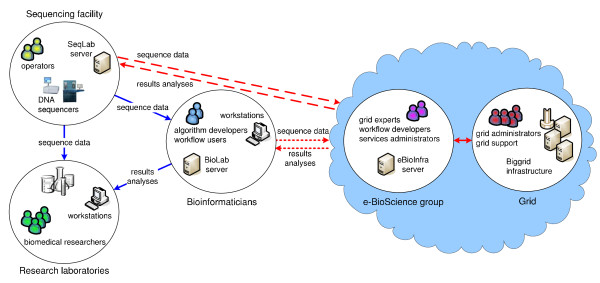
**People, resources and data flow in the platforms for DNA sequencing**. Blue arrows: data flow on local infrastructure. Data is transferred from the SeqLab to the BioLab server and results are downloaded from the BioLab server. Red dotted arrows: The VBrowser is used for data transfer between servers and grid resources and for running workflows on the grid. Red dashed arrows: Data can be transferred directly from the SeqLab server to the grid.

The informatics resources adopted in this process currently include four major components as illustrated in Figure 1: a server at the sequencing laboratory (SeqLab); the researcher's workstation(s), for interactive analysis and remote access to the servers; a server for bioinformatics analysis (BioLab); and the bioinformatician's workstation(s).

Data analysis may vary for each experiment, but it often includes the following steps. The sequences are retrieved from the SeqLab server and stored on the BioLab server. The data requires conversion (file reformatting), after which it can be further processed by the other components of the analysis pipeline. In many cases multiple samples are pooled together in one sequencing experiment. One or more nucleotide labels (also called multiplex labels or bar codes) are added to the sequence fragments and have to be identified afterwards to group all the sequences belonging to one sample. The sequences are identified by aligning them against a reference database, such as GenBank [13]. The inexact matching (alignment) is performed with the Basic Local Alignment Tool (BLAST) [14], the BLAST Like Alignment Tool (BLAT) [15] or the Burrows-Wheeler Aligner (BWA) [16]. Different databases may be used depending on the application. The alignment results are summarized to give information about, for example, genes, exons and species in the samples. When the analysis is complete, the results are returned to the researcher via a password protected web-interface or FTP server.

Since specific applications may require customized reference databases, it is necessary to have a local installation to run BLAST or other alignment software. Even for standard BLAST searches it is neither desirable nor possible to overload public BLAST services as, for example, provided by the EBI [17] and NCBI [18]. Since we were confronted with long computation times and the large size of (temporary)files, we needed to increase our computational capacity.

In addition, an increase of in-house sequence data is expected for which we need to build new application specific pipelines and where custom and third party software has to be (re)used. Therefore we also had to manage the analysis pipelines in a more efficient way. Various researchers collaborate simultaneously inside and outside our institution, so it is important to manage the access rights to the tools and the data properly. To summarize, an infrastructure was needed where we can access different systems with the same interface, manage and run the pipelines in a structured way, distribute the analyses results and where the user has access to large data storage and computing capacity. Most of the above mentioned issues were addressed by adopting and extending the e-BioScience infrastructure available in our organization.

### e-BioScience infrastructure

The e-BioScience Infrastructure (e-BioInfra) provides access to federated compute and storage capacity and data management facilities. It has its origins in the subprogram Medical Diagnosis and Imaging of the VL-e project (VLEMED), being initially developed to facilitate storage and analysis of functional MRI data [11,12]. The high-performance resources are provided by the DutchGrid [6]. All clusters run Scientific Linux and gLite grid middleware [19], being fully compatible with European Grid projects such as EGEE [20] and EGI [7]. Among other services, the infrastructure provides a gLite Logical File Catalog (LFC) server to federate data storage and a Virtual Organization Membership Service (VOMS) for authorization. Users need to possess a valid X509 certificate and become members of the LSGRID or VLEMED Virtual Organization (VO) to have access to the grid resources and the e-BioInfra services.

The e-BioInfra platform is based on the user front-end provided by the Virtual Resource Browser (VBrowser) [21,22] and adopts a Service Oriented Architecture (SOA). The main functions are performed by services that run on the e-BioInfra server, among others the communication with lower level grid resources. Most of the available services (e.g., workflow management and job monitoring) are called transparently from the VBrowser client that runs on the user's workstation, hiding complex de-tails from the end-user. A detailed description of the e-BioInfra can be found in Olabarriaga *et al *[11]. For completeness a short summary is presented below.

**Figure 2:**
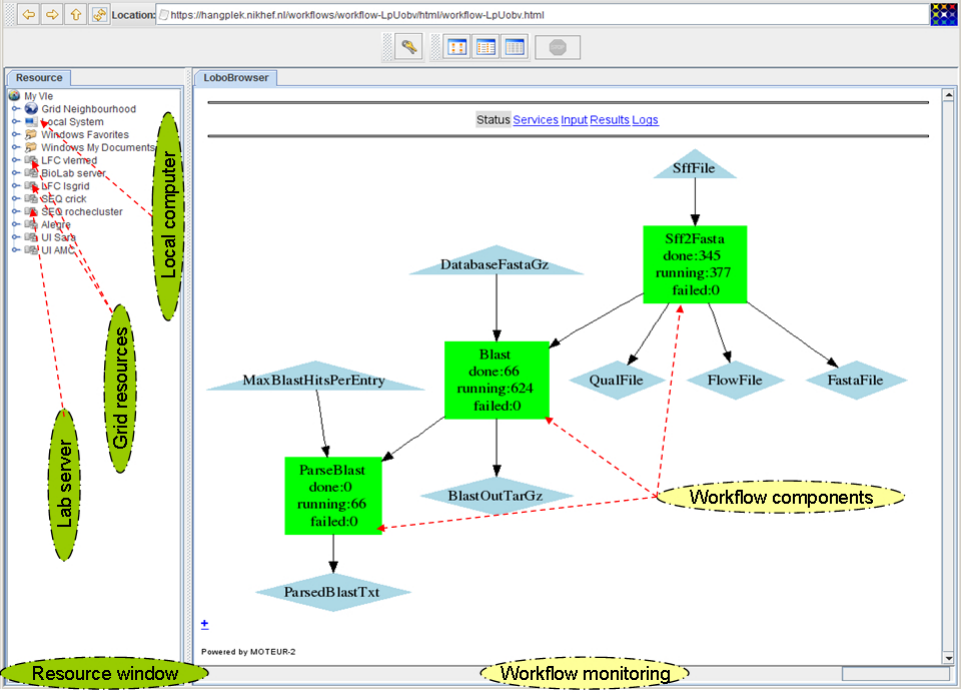
**Screenshot of the VBrowser GUI**. Screenshot of the VBrowser GUI showing the resource (left side) and the workflow (right side) windows. The resource window illustrates storage locations at the grid, remote servers and local computer (green ellipses). The color of the workflow components indicate the status of the jobs; purple components are completed, red are failed and in green still running.

The VBrowser is a tool developed in the VL-e project that provides access to local and grid resources (Figure 2) using similar look-and-feel as popular file explorer interfaces. Using grid proxy authentication, the VBrowser supports various grid, remote and local file systems (e.g. LFC, Grid-FTP and secure FTP) and presents them to the user similarly. Additionally, it is an extensible platform that allows extra plug-ins to be installed to enhance the browsing capabilities, for example for workflow execution with MOTEUR and grid job monitoring.

The MOTEUR workflow management system [23] is used to run workflows on grid infrastructures, providing a flexible workflow description and grid enactment framework. The MOTEUR engine is activated from a web service interface and the corresponding VBrowser plug-in. Workflows are described in the Simple Concept Unified Flow Language (SCUFL) of the Taverna workbench [24]. Iteration operators describe one-to-one (dot product) and all-to-all (cross product) data combination and indicate how to combine the input values given to the workflow (e.g., for parameter or data sweeps). The workflow components are Linux executables that are wrapped on the fly into grid jobs by the Grid Application Service Wrapper (GASW) [25], based on an XML document (GASW descriptor) that indicates the components' inputs, outputs, executable and software dependencies.

To run workflows a user opens the SCUFL document on the VBrowser, specifies the input parameters (e.g. names of data files to analyze), and starts the workflow. The SCUFL, inputs and user grid proxy are sent to the MOTEUR service, which runs the workflow by executing grid jobs for each of the workflow tasks. The status of the workflow is monitored from a web interface and the progress of individual jobs can be monitored with the job-monitoring plug-in. Once the workflow is completed, the user can interactively inspect the results stored on grid resources with the VBrowser.

### Grid infrastructure for sequencing

In the following sections we describe how the e-BioInfra was used to implement grid-based analysis of sequence data. The sequence analysis programs were wrapped as workflow components and combined into workflows that are handled by the MOTEUR service. All data and programs are stored on the LFC.

### File Management

All files (data, programs and workflows) are stored in the LFC server of the LSGRID and VLEMED VO. The analysis tools and workflows are stored in a separate directory that is readable to all members of these VOs and organized into the following sub-directories:

• Scufl: workflow descriptions;

• Gasw: description of workflow components for MOTEUR;

• perlScripts: Perl scripts implementing workflow components;

• bin: linux binaries implementing the analysis tools executed by the workflow components.

• shFiles: Linux shell scripts implementing workflow components;

All files that belong to a study (data, results) are stored in a separate directory under control of the user. The structure of the data directories is fixed, separating inputs from output results generated by the workflow. Each workflow component stores outputs in a separate subdirectory using unique file names that are automatically generated for each workflow run. Before running workflows, the user needs to create a directory on the LFC, copy the sequence data to it and change the access rights if data protection is required. All necessary subdirectories to store output data are created automatically during the workflow execution. The data transfer between the SeqLab and BioLab servers to the grid storage is performed interactively by the user with the VBrowser. After workflow execution the results are transferred to the BioLab server where they are distributed to the biomedical researcher via a password protected website or directly to the SeqLab server (red dotted and dashed arrows in Figure 1).

### From executables to gridified workflow components

New workflow components were developed for DNA sequence data analysis. These include file format conversion (based on the sffinfo conversion tool of the Roche package), sequence alignment (BLAST and BLAT), and a best-hit selection of the Blast results (a custom perl script). Note that the executables must be compatible with the system architecture of the grid nodes (During the writing, the infrastructure was being upgraded from 32 to 64 bits Scientific Linux 5, requiring upgrade of some binaries).

Each of the executables is activated from a Perl script originally designed to execute on a local Linux server from the command line. The scripts manage the in- and output files, dependencies from other executables and libraries, VO environment, and they call the executable using the proper command line arguments.

Before converting these scripts into gridified workflow components, it was necessary to streamline the input, output and error control. All hard coded paths, files and other parameters were removed from the script. These parameters are now given to the script via the command line. This allows for a flexible definition of the input files and automatic generation of output files or directory names. The script generates error messages when particular functions can not be executed. These error messages are stored in a file that can be examined by the user when something goes wrong during execution of the workflow.

To convert the scripts into workflow components for MOTEUR, a GASW descriptor had to be created. It specifies the name of the script, the in- and outputs for the command line generation and external software and hardware dependencies. To facilitate this process we have developed a perl script that automatically generates the GASW and a SCUFL file to execute one component. The GASW descriptor includes parameters to indicate the name of the output directories and unique file naming. This was used in the following manner: each workflow component writes the output in a subdirectory of the input data location. The output files are generated with unique file names by adding random numbers to the given output filenames. This simplifies the execution of the workflow, because the user does not have to worry about overwriting previous results.

### All Steps

**Figure 3:**
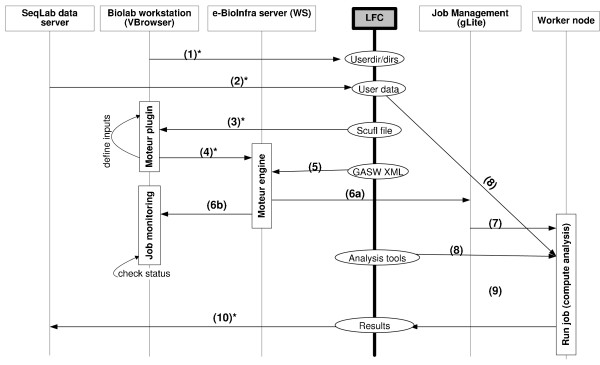
**Data management of sequencing analysis**. (*) indicates users action (1) create directory structure, (2) copy analysis data, (3) select workflow, (4) run workflow, (5) GASW description, (6a) submit job, (6b) monitor jobs, (7) start job, (8) download data/analysis tools, (9) upload results, (10) copy results.

The steps necessary to perform data analysis using this methodology are visualized in Figure 3. Each step with a number is described below and corresponds to an action performed by the user or done automatically by the services. For clarity, many of the automatic steps that are transparently carried out by the e-BioInfra components are omitted in the figure and in the explanation. More details can be found in Olabarriaga *et al *[11].

The directory structure is created by the end-user with own credentials on the LFC server (1). The input data for the analysis applications are copied to the users private or shared directory (2). This data is given as input in the MOTEUR plug-in window (3) after selecting the workflow SCUFL file with the VBrowser. Multiple sequence data can be aligned against multiple databases at the same time by providing lists of input values. The plug-in then activates the MOTEUR service (4), which handles the input combinations indicated by cross/dot product and runs all the necessary tasks. It downloads the GASW descriptor (5) and generates the wrapper shell script that downloads the input files from the grid storage, executes the script file with the given parameters, and uploads the output files to the grid storage. This shell script is executed as a job by MOTEUR using the gLite middleware (the job description language file (JDL) is also generated automatically). Each job is then submitted (6a) to the gLite Workload Management System (WMS) and eventually started (7) on a worker node, where the analysis tools and data are downloaded (8) and the task is executed. The progress of the workflow is automatically updated in the workflow monitoring window (in Figure 3). The status of the submitted jobs can be monitored in the job monitoring window (6b). When the analysis is completed, the output result is automatically uploaded (9) to the LFC server in a specified directory. These results can be the input for the next component of the workflow, being handled again by MOTEUR until the workflow execution has finished completely. The output data can be browsed, viewed and copied (10) interactively by the user with the VBrowser.

## Results

### Workflows

The components for sequence analysis were combined into workflows (Figure 2) using the editor of the Taverna workbench. The workflows were uploaded in the community repository provided by the myexperiment project [26-28]. We created a workflow with 3 components: data conversion (sffTo-Fasta), alignment to a given reference database (Blast) and selection of the BLAST results (Parse-Blast) [26]. This workflow was used as a basis for the second workflow, where the alignment tool was replaced by Blat and where the ParseBlast component was removed [27]. We also combined the two workflows by adding the BLAT component in the original workflow, enabling the user to perform the alignments with both programs in one run for comparison [28]. The sequence data and reference databases are selected by the user at run-time. The iteration operators are used to combine the input data and the reference database, indicating that the workflow should be executed for all combinations automatically.

### BLAST Workflow Evaluation

To evaluate the behavior and performance of the developed workflows on the Dutch grid, we executed them for data acquired as part of a virus discovery study. The sequences need to be aligned against several database sections of Genbank, and those that do not have a match in Genbank represent possible new viruses that can be further analyzed. For the alignment step we have used the Blast workflow. The workflow was run several times for different input data, i.e. all the sequence experiments of the virus discovery study, to check the success rate and the execution time. Each experiment contains several samples that can vary in size. We have aligned 722 samples from 15 sequencing experiments against two reference databases: the viral section of Genbank (version 175, 125 MB) and against human ribosomal sequences in Genbank (version 175, 752 KB). Therefore BLAST was executed twice for each sample set. Additionally we also executed the workflow aligning all 722 samples at the same time against the two databases. Additional file 1 summarizes the results, presenting the data size for each experiment, the elapsed time of the workflows and the success rate. At this point we have not yet optimized the size of the query sequences for an optimal execution time.

In total 1444 samples containing 4,783,684 sequences were aligned to two databases by the execution of 16 workflows in the period of August-September 2010. The total CPU time for all the individual experiments ("A-O" in Additional file 1) was 413 hours (data not shown), representing approximately 17 CPU days of computing effort if performed sequentially. The total elapsed time to run the single workflow for all samples ("All" in Additional file 1) was less than 14 hours, representing a speedup of around 30 under the conditions of the grid infrastructure in that period. The workflows successfully performed 7177 jobs that consumed and generated 383 GB of disk space on the worker nodes.

An evaluation of success and failure should be done from different perspectives.

From the end-user point of view, success is measured by the number of generated results compared to the expectations. As shown in Additional file 1 the overall success rate is rather high, in average 99.2 ± 1:2% ∈ [96.3, 100]%. In many cases (9/16 workflows), 100% success was obtained, which means that the alignment could be accomplished in most cases without further user intervention than starting the workflow.

From the grid execution point of view, success is determined by the jobs that are submitted to perform the task of each workflow component with the given combination of inputs. Although each "task" roughly corresponds to one "job", the number of submitted jobs can be larger because of the built-in retry mechanism in MOTEUR. Therefore we also present data about the success rate considering the number of grid jobs that were submitted and monitored by MOTEUR. Note that the success rate is lower when considering grid execution, in average 98.7 ± 1.3% ∈ [95.0, 100]%, and that in only 4 of the workflows 100% success was achieved, namely when a small number of samples were processed. However, in most cases the workflow manager was able to retry the jobs and generate the result without user intervention.

### Related work

Sequence alignment programs such as Blast and Blat have become popular use cases for grid computing applications because they can easily benefit from data parallelization. Blast and other bioinformatics applications have been ported to desktop grids [29,30], clusters [31,32] and (inter)national grids [33-35]. Strategies to optimize the fragmentation of the database and/or the query sequences are described in Mirto *et al *[35]. Trombetti *et al *[33] described a procedure to automatically download and manage Blast databases on grid storage elements. Also in other areas there is much interest for the execution of large experiments on grids, for example in virtual protein docking [36] and DNA transformation [37].

Regarding platforms for running applications on grids, the number of comparable initiatives, for sequencing and many other bioinformatics applications is too large to list here. The problems being faced by the data explosion in the Bioinformatics field have been addressed by a large number of international projects and organizations, giving rise to various platforms that are mostly adopted inside the project or organization. Some notable examples of generic systems that combine workflow and grid technology are the P-Grade Grid Portal [38] and the Grid Workflow Execution Service (GWES) [39]. The P-Grade Grid Portal supports the execution of workflows and workflow-based parameter studies on various grid infrastructures. GWES is the workflow enactment engine implemented for the Fraunhofer Resource Grid. It coordinates the composition and execution process of grid or Service Oriented Architecture (SOA) workflows, also providing interfaces to a Web Portal, a command line client for user interaction, and low-level grid middleware for the invocation of application operations. Taverna [24] is a platform that is widely used in the bioinformatics community for the development and execution of data analysis workflows. Local tools and remote web services can be incorporated into one workflow. Previous versions of the Taverna workbench were not grid enabled, but plug-ins now allow for workflow execution on grids in more recent versions. Another framework, G-Eclipse [40], allows users and developers to access computing grids and cloud computing resources in a unified way. The framework itself is independent from a certain grid middleware or cloud computing provider. More recently, Callaghan *et al *[41] described the Pegasus Workflow Management System in combination with a logging system to analyze the performance of workflows. The system is able to automatically resubmit failed jobs by executing a new workflow without resubmitting jobs that are already finished successfully.

## Discussion

In this pilot project we reused an infrastructure described in Olabarriaga *et al *[11] that was setup for medical image analysis for applications in bioinformatics, using sequence alignment as test cases. We decided to use it because it fulfills our requirements regarding workflows and Grid technology, as well as there was much in-house expertise about this platform. This facilitated tremendously our first steps into this new technology.

The choice for Grid technology was motivated both by the need of access to more storage and computing power and the wish to facilitate the exchange of data and analysis methodologies captured into workflows with collaborators around the globe. Workflow technology was chosen to speed up the implementation of data analysis pipelines, since it facilitates reuse of software (workflow) components, automatically enables their execution on grids, and enables their straightforward replacement by new or alternative algorithms and implementations. By using a generic layer of tools built on top of grid middleware, we could focus on the development of workflows to perform the specific data analysis methods required to face the challenges and ambition of the high-throughput sequencing facility at the AMC. This generic layer has protected the bioinformatician and the e-scientist from frequent changes on the grid infrastructure, which most of the time cause disruption to the execution of grid applications. During the project there were major changes, several upgrades and expansions on the Dutch grid infrastructure. Although the e-BioInfra services had to be adapted to these changes, the workflows remained essentially the same. The users only perceived an increase in capacity and improved stability of the infrastructure as a whole. In the absence of such a high-level platform, individual jobs need to be submitted and monitored by the users using command-line gLite utilities, either manually or via home-made scripts, which is time consuming and more prone to error when the number of jobs grow. Moreover, the wheel has to be reinvented for each different experiment to be performed on the grid, whereas in our set-up much knowledge can be reused by adapting the workflows and their components.

In practice problems can be encountered when running workflows on a production grid. This is a well-acknowledged fact in the context of distributed computing infrastructures, and the Dutch grid is no exception. Jobs failed due to various reasons, for example due to maintenance or misconfiguration of the grid nodes, incorrect functioning of the grid middleware service, or because of large traffic on grid resources. In particular data transfer has not been optimized yet in our workflows, causing many jobs to fail due to transfers timeout. During development we also came across other problems such as the inability to share workflows initially because the access rights to the software files were set too strict. We also faced problems respective to the lifespan of grid certificate proxies, which by policy are short-lived and expire before all jobs can be finished. It is also important to realize that the grid and workflow management systems are still under development, and still prone to failure. Under such failure conditions, support from the grid and workflow experts is still needed to handle possible runtime problems. This support was provided by a team of experts that develop and run the e-Bioinfra platform. However, more automated and controllable failure management is necessary to facilitate the execution of large data analysis experiments on complex infrastructures such as grids.

The success rates reported here are high considering other statistics published by studies that used the EGEE infrastructure (e.g., Glatard *et al *[42]), but it indicates that jobs occasionally fail, which is a known problem on production grid infrastructures. The tasks that fail, even after automatic resubmission, need to be submitted again. At the moment the user needs to select these jobs manually, but in the future we would like to automate this process. Callaghan *et al *[41] describe an infrastructure that automatically detects failed jobs and starts a new workflow for these jobs. In our case we would like to choose whether the jobs need to be resubmitted, because errors can occur on the application level, for example when too much memory or disk space is used. Overall, the success rate is acceptable, however, a procedure to automatically detect what jobs need to be resubmitted is desirable.

Concerning usability, currently the analyses are performed by the bioinformatician, who also handles file transfer to/from the grid manually. Ideally the full analyses would be performed by the biomedical researcher directly with the VBrowser, which has a reasonably user-friendly interface and can be used with minimal instruction. Another option being investigated is to adopt a web interface, however the data management and access control still present challenges. Both alternatives have been evaluated for medical imaging applications already, and we expect that the results are also applicable for bioinformatics.

Proper handling of data access control is also important to ensure that (sequence) data in a Grid environment can not be accessed by unauthorized persons. Currently the user who uploads sequencing data must be aware of all details about access control rights on grid storage, including the LFC and lower level services. Considering the usage model adopted on grids, where the members of a virtual organization are "trusted", users need to understand that in principle all files are accessible to all members of the same VO. It is necessary to invest in a good procedure to protect sensitive data, but at the same time allow other data and workflows to be shared within the community.

Improvements on the workflow and component description are also desirable. At the moment it is not possible to create grid workflow components that have an unknown amount of input or output parameters. For example, a component that takes a compressed file as input and unknown amount of output files can not be described using the current GASW implementation. This is a problem faced by other applications as well. At the moment we are investigating how to handle this situation with other types of workflow components (e.g., BeanShell).

## Conclusions and future work

In this paper we described the grid-based workflow approach adopted to face the challenge of analyzing an increasing amount of data generated by modern sequencers. Our choice for using the e-BioInfra platform was based on the compatibility with the Dutch Grid infrastructure, the general applicability of the system and the in-house knowledge about it. When getting started with cyberinfrastructures from an unrelated field, it is useful to get support on different levels of the infrastructure to setup grid workflows for bioinformatics in an efficient way.

As a pilot case we explored simple data analysis pipelines that perform sequence alignment. The workflows are available on the myExperiment website [26-28] and the e-BioInfra website [43] and can be reused by other bioinformaticians. Evaluation results showed that the total time required to perform the alignment can be drastically reduced with this approach. Although in this work we only presented results for the BLAST method, our goal was not to optimize this particular method but to investigate if a Grid/workflow-based framework can be made sufficiently flexible and generic to be used for various bioinformatics applications in a production environment with large computational capacity. The pilot study showed that this is possible.

With the knowledge gained in this pilot project the implementation of other bioinformatics workflows became easier and faster. Workflows can be easily shared with members of the same virtual organization (LSGRID and VLEMED in this case) and the use of Grid also ensures that the workflows are scalable with respect to required compute power. By using the e-BioInfra platform, the execution time could be reduced to a few hours regardless of the amount of input data, given that the input data is split and processed in parallel. The amount of data used in this study is relatively easy to manage, but for other DNA sequencers, like the ABI Solid or Illumina Hiseq, the increasing amount of data becomes more problematic. Moreover, when a new workflow is implemented we would like to run it on all earlier sequence experiments. Here we can really benefit from data parallelism. For example, in a current project we are performing a pair-wise comparison between 400,000 sequences produced in a single experiment. When this is run serially on our regular server this would take approximately 3.5 days, and this time will grow exponentially as the throughput of the DNA sequencers increases. For these types of sequencing projects we are planning to optimize the splitting of the experiment data, experiment with different alignment programs and test different parameter settings.

Currently the e-bioinfra is used almost daily by bioinformaticians in our group that work on sequence analysis. New workflows are being developed with other alignment programs and other analysis tools are implemented as workflow components. Also in the field of proteomics and metabolomics workflows have been developed and used, although these are still at an experimental phase. The e-BioInfra has the potential to extend the toolbox for bioinformaticians with state-of-the-art computing resources and the possibility to run workflows on the grid from one single front-end.

Finally, in this pilot study we observed that besides the hardware and software tools the expertise about developing workflows and working with cyberinfrastructures is needed, because these technologies are not trivial. People experienced with grid infrastructures and workflow systems are needed, because many things can go wrong along the way. Inhouse expertise on grid and workflow technology was necessary for successful integration of e-science approaches into daily work.

## Authors' contributions

AVK and SDO conceived the study. AL, BVS and SDO designed the workflows. AL and BVS implemented and tested the workflows. MDV and FB performed the sequence data acquisition and contributed to the design of the workflows. All authors read and approved the final manuscript.

## Supplementary Material

Additional file 1 **Workflow runtime and success rate**. Performance data for workflow executions (see text). Each row indicates one workflow execution (Exp.) for the given number of samples (# Sam) containing the number of sequences (# Seq). For each workflow component (Sff2Fasta, Blast, ParseBlast), the number of tasks is presented (successfully finished/submitted). Although each task roughly corresponds to one job, the number of submitted jobs can be larger because of the built-in retry mechanism in MOTEUR. For workflow as a whole, the percentage of success is calculated by dividing the number of generated results (output of the ParseBlast component) by the number expected results (in this case 2 × # Samples). For the grid execution, the success is calculated by dividing the number of successful by the number of submitted jobs. The elapsed time is the total time from starting the workflow until the end of its execution. This information was extracted from the monitoring information from the workflow management service. Click here for file
