# Supplementary material for: Initial steps towards a production platform for DNA sequence analysis on the grid
Source: BMC Bioinformatics. 2010 Dec 14;11:598. doi: 10.1186/1471-2105-11-598 (PMC3018473; doi:10.1186/1471-2105-11-598)
Supplement: Additional file 1 — Workflow runtime and success rate. Performance data for workflow executions (see text). Each row indicates one workflow execution (Exp.) for the given number of samples (# Sam) containing the number of sequences (# Seq). For each workflow component (Sff2Fasta, Blast, ParseBlast), the number of tasks is presented (successfully finished/submitted). Although each task roughly corresponds to one job, the number of submitted jobs can be larger because of the built-in retry mechanism in MOTEUR. For workflow as a whole, the percentage of success is calculated by dividing the number of generated results (output of the ParseBlast component) by the number expected results (in this case 2 × # Samples). For the grid execution, the success is calculated by dividing the number of successful by the number of submitted jobs. The elapsed time is the total time from starting the workflow until the end of its execution. This information was extracted from the monitoring information from the workflow management service. [file 1471-2105-11-598-S1.PDF]

Table 1: Performance data for workflow executions (see text). Each row indicates one workflow execution (Exp.) for the given number of samples (# Sam) containing the number of sequences (# Seq). For each workflow component (Sff2Fasta, Blast, ParseBlast), the number of tasks is presented (successfully finished / submitted). Although each task roughly corresponds to one job, the number of submitted jobs can be larger because of the built-in retry mechanism in MOTEUR. For workflow as a whole, the percentage of success is calculated by dividing the number of generated results (output of the ParseBlast component) by the number expected results (in this case 2 $\times$ # Samples). For the grid execution, the success is calculated by dividing the number of successful by the number of submitted jobs. The elapsed time is the total time from starting the workflow until the end of its execution. This information was extracted from the monitoring information from the workflow management service.

**Exp**	**# Sam**	**# Seq**	**Workflow Tasks**			**Workflow Results**			**Jobs**			**Time (hrs)**
			**Sff2Fasta**	**Blast**	**ParseBlast**	**Ok**	**failed**	**success**	**Ok**	**failed**	**success**	
A	96	37,632	95/96	190/190	189/190	190	3	98.4	474	10	97.9	4.2
B	44	2,338	44/44	88/88	88/88	88	0	100	220	0	100	3
C	48	149,949	48/48	96/96	95/96	96	1	99.0	239	3	98.8	3.2
D	93	205,258	93/93	186/186	186/186	186	0	100	465	7	98.5	10.5
E	12	36,721	40524	24/24	24/24	24	0	100	60	0	100	4.2
F	45	13,974	45/45	89/90	89/89	89	1	98.9	223	3	98.7	4.8
G	24	34,541	24/24	48/48	48/48	48	0	100	120	0	100	3.2
H	45	9,096	45/45	90/90	90/90	90	0	100	225	1	99.6	3
I	27	7,463	26/27	52/52	52/52	52	2	96.3	130	3	97.7	3
J	54	474,821	54/54	106/108	105/106	106	3	97.2	265	14	95.0	4.6
K	53	504,277	53/53	106/106	106/106	106	0	100	265	5	98.1	3.7
L	55	383,796	55/55	110/110	110/110	110	0	100	275	2	99.3	3.5
M	56	368,975	56/56	112/112	112/112	112	0	100	280	8	97.2	3.8
N	56	65,749	55/56	110/110	110/110	110	2	98.2	275	5	98.2	3
O	14	97,252	14/14	28/28	28/28	28	0	100	70	0	100	2.8
All	722	2,391,842	719/722	1436/1438	1436/1436	1436	8	99.4	3591	12	99.7	13.7
Total	1444	4,783,684	1438/1444	2871/2876	2868/2871	2871	20	99.3	7177	73	98.9	74.2
